# Safety, effectiveness, and adherence of a short and all-oral treatment regimen for the treatment of rifampicin-resistant tuberculosis in Niger: a study protocol of a pragmatic randomised clinical trial with stratified block randomisation

**DOI:** 10.1186/s13063-022-06912-7

**Published:** 2022-12-13

**Authors:** Mahamadou Bassirou Souleymane, Tom Decroo, Alphazazi Soumana, Ibrahim Maman Lawan, Assiatou Gagara-Issoufou, Souleymane Halidou-Moussa, Nimer Ortuño-Gutiérrez, Eric Adehossi, Saïdou Mamadou, Armand Van Deun, Alberto Piubello

**Affiliations:** 1 Damien Foundation, Niamey, Niger; 2 Institute of Tropical Medicine, Antwerp, Belgium; 3 Research Foundation Flanders, Brussels, Belgium; 4 Programme National de Lutte contre la Tuberculose, Programme, Niamey, Niger; 5 Université Abdou Moumouni de Niamey, Faculté des Science de la Santé, Niamey, Niger; 6 Hopital National Amirou Boubacar Diallo, Pneumo-phtysiologie, Niamey, Niger; 7 Damien Foundation, Brussels, Belgium; 8 Independent Consultant, Leuven, Belgium

**Keywords:** Rifampicin resistance tuberculosis, All oral treatment, New anti-tuberculosis drugs, Niger

## Abstract

**Background:**

Rifampicin-resistant tuberculosis (RR-TB) treatment requires combination treatment, which frequently causes serious adverse events and globally results in not much more than 60% treatment success. In Niger, a high cure rate was obtained with a RR-TB treatment strategy based on a second-line injectable drug (SLID)-containing Short Treatment Regimen (STR), with linezolid replacing the SLID in patients with ototoxicity. Given the availability of novel anti-tuberculosis drugs, WHO recommends all-oral RR-TB treatment. Considering the high level of success with the Niger treatment strategy, it would only be justified to replace it in case robust evidence shows that the WHO all-oral bedaquiline/linezolid (BDQ/LZD)-containing STR (experimental arm) performs better than the Niger RR-TB treatment strategy, (control arm) in terms of safety, effectiveness and adherence.

**Methods:**

A pragmatic randomised clinical trial (RCT) using stratified block randomisation, conducted between April 2021 and March 2024, prospectively enrols participants diagnosed with RR-TB in one of the four RR-TB units of the nation. Depending of the month in which patients are diagnosed with RR-TB, patients with FQ-susceptible RR-TB are enrolled in either the experimental arm or control arm.

**Discussion:**

To increase the feasibility of conducting a RCT, embedded in routine activities of all Niger’s RR-TB Units, we used a creative trial design. We randomised by monthly blocks, whereby the regimen used changes every month, using the month of RR-TB diagnosis as stratifying variable. This approach was deemed feasible for Niger's national tuberculosis programme, as it simplifies the work of the clinicians running the RR-TB units. Our creative design may serve as an example for other national programs. Findings will inform national and international RR-TB treatment guidelines, and will also strengthen the evidence-base on how to develop robust RR-TB treatment regimens.

**Trial registration:**

Pan African Clinical Trial Register PACTR202203645724919. Registered on 15 March 2022.

## Administrative information

Note: the numbers in curly brackets in this protocol refer to SPIRIT checklist item numbers. The order of the items has been modified to group similar items (see http://www.equator-network.org/reporting-guidelines/spirit-2013-statement-defining-standard-protocol-items-for-clinical-trials/).

**Table:** 

**Title {1}**	Safety, effectiveness, and adherence of a short and all-oral treatment regimen for the treatment of rifampicin-resistant tuberculosis in Niger: a study protocol of a pragmatic randomised clinical trial with stratified block randomisation
**Trial registration {2a and 2b}.**	Retrospectively registered under N° PACTR202203645724919 in Pan African Clinical Trial Registry
**Protocol version {3}**	11/02/2021 version 4
**Funding {4}**	Damien Foundation Global Found Niger grant IMT/DGD The funding source had no role in the study design; in the collection, management, analysis, or interpretation of data; in the writing of the report; or in the decision to submit the report for publication.
**Author details {5a}**	^1^Damien Foundation Niger ^2^Institute of Tropical Medicine Antwerp ^3^National TB Programme Niger
**Name and contact information for the trial sponsor {5b}**	Damien Foundation and ITM are the sponsors of this trial
**Role of sponsor {5c}**	The sponsors are involved in all trial activities, including study design, data collection and analysis, and writing/submission of reporting and scientific publications.

## Introduction

### Background and rationale {6a}

The treatment of rifampicin-resistant tuberculosis (RR-TB), TB resistant to the most powerful anti-TB drug, requires a more complex regimen than the treatment of rifampicin-susceptible TB and more frequently causes severe adverse drug reactions. Until 2016, 18–24 months long and poorly tolerated RR-TB treatment regimens were recommended [1]. These regimens were individualised, thus not standardised, and resulted in not much more than 60% treatment success [2].

In 2016, WHO recommended a standardised short treatment regimen (STR) for patients not yet exposed to second-line drugs and without resistance to the main components of this STR [3]. This STR had achieved a high cure rate (over 80%) among RR-TB patients in multiple programmatic settings [4–8]. However, this STR included a second-line injectable drug (SLID; amikacin, kanamycin or capreomycin), known for causing severe hearing loss as main adverse drug reaction [9]. A review showed that SLID had to be discontinued in about 10% of patients due to adverse events (AE) [10].

The increasing availability of novel drugs, such as bedaquiline (BDQ) and delamanid (DLM), and repurposed drug, such as linezolid (LZD) and clofazimine (CFZ), allow the designing of potent RR-TB treatment regimens solely relying on oral drugs. The 2019 WHO RR-TB treatment guidelines recommend replacing the STR’s SLID with bedaquiline [11]. WHO also recommends research on STR using both BDQ and LZD in patients who have not yet taken second-line drugs for more than one month and for whom fluoroquinolone resistance has been ruled out [12]. This WHO BDQ/LZD-containing all-oral STR resulted in 73% end-of-treatment success in South Africa [13, 14], considerably lower than 83% relapse-free cure obtained with the RR-TB treatment strategy used in Niger [8].

In Niger, patients are treated with a SLID-containing STR. The STR’s SLID is replaced by LZD in patients with contraindications for SLID, mainly ototoxicity. Audiometry is done at baseline and on a monthly basis during treatment. As any audiometry abnormality serves as an indication for switching to LZD, not a single RR-TB patient developed severe hearing loss since 2017 [15]. BDQ is safeguarded for the rare patients with treatment failure or relapse after a first STR, leaving not a single patient without a potentially successful retreatment option [8].

Considering the highly effective RR-TB treatment strategy used in Niger, it was not justified to introduce the WHO BDQ/LZD-containing STR without a rigorous evaluation. We therefore aim to compare the safety, effectiveness and adherence of the WHO all-oral BDQ/LZD-containing STR (experimental arm) with the Niger RR-TB treatment strategy, using a SLID-containing regimen, with LZD replacing the SLID in case of ototoxicity (control arm). To provide high-level evidence on the one hand, but also timely inform local and international guidelines, we used an approach that would be feasible for the local RR-TB programme. We designed a pragmatic randomised clinical trial with stratified block randomisation, using the month of RR-TB diagnosis as stratifying variable. The study protocol is presented here.

### Objectives {7}

The primary objective of the trial is the comparison of safety (any grade 3–5 AE) between the experimental arm (WHO all-oral regimen) and the control arm (Niger RR-TB treatment strategy: a STR with either a SLID or LZD, depending on the presence of audiometry abnormalities).

Secondary objectives include:

1. Describe the effectiveness (“therapeutic success” being defined as “cure” or “treatment completed” without relapse 12 months after the end of treatment) of the two treatment arms, and estimate predictors of effectiveness
2. Determine adherence to the regimen (assessed by measuring the proportion of patients who took at least 90% of the doses of the RR-TB treatment regimen) of the two treatment arms.

## Methods

We present all items defined by the Standard Protocol Items: Recommendations for Interventional Trials (SPIRIT) statement.

### Trial design {8}

This is a pragmatic randomised clinical trial using stratified block randomisation to compare the safety, effectiveness and adherence of an experimental arm, the WHO all-oral regimen, including BDQ and LZD for all, with the control arm, the Niger RR-TB treatment strategy, using a STR that contains either a SLID or LZD, depending on the presence of audiometry abnormalities. We hypothesise that the STR that contains either a SLID or LZD is more safe, thus superior, than the WHO all-oral regimen, with LZD for all.

### Study setting {9}

Since 2008, RR-TB care in Niger is provided by the National Tuberculosis Programme (NTP), supported by a Belgian NGO, the Damien Foundation (DF), and the Antwerp Institute of Tropical Medicine (ITM).

The country has 20 Xpert MTB/RIF machines (used for molecular RR-TB diagnosis) distributed over its eight Regions. Drug susceptibility testing (DST) for fluoroquinolone and SLID relies on second-line line probe assay (LPA) and culture-based phenotypic DST on solid medium, carried out at the National Referral Laboratory of Niamey (NRL). The NTP applies an exhaustive and systematic quality assurance assessment for all the different types of laboratory tests (sputum smear microscopy, culture and DST). Samples are routinely sent to the ITM in Antwerp for phenotypic and genotypic DST and spoligotyping.

Patients are treated in the country’s four RR-TB units (Niamey, Maradi, Zinder and Tahoua), managed by the NTP, and in close collaboration with DF. RR-TB care is ambulatory, except for bedridden patients who are hospitalised in the referral structures of the four cities. A comprehensive package is provided, including nutritional support, transport costs for patients to reach the RR-TB units, treatment for AE and home visits. Active drug safety monitoring (aDSM) is in place and produces regular reports.

### Eligibility criteria {10}

Patients are eligible for the study if all of the following conditions are met: (1) have bacteriologically confirmed TB with evidence of resistance to rifampicin (for children the diagnosis is not always bacteriologically confirmed as the RR-TB diagnosis may be based on clinical TB diagnosis plus history of a close contact with a confirmed RR-TB case) and susceptibility to fluoroquinolones and (2) be willing and able to give informed consent to participate in the study (signed or witnessed consent if the patient is illiterate; signed or witnessed consent by a child’s parent or legal guardian).

Exclusion criteria: a patient is ineligible for the study if any of the following conditions are present: (1) previous treatment for more than one month with second-line TB drugs included in the treatment regimens to be evaluated (disregard E and Z, which may have been used previously in first-line treatment), (2) a health rate-corrected QT (QTc) interval ≥ 500 msec at baseline that is not corrected by medical management, (3) any other medical contraindications for taking the study regimens, (4) being younger than 6 years old, and (5) diabetes mellitus.

As during routine practice, pregnant women are not excluded from treatment and thus not from the study. The patient information sheet states that there is a lack of safety data during pregnancy.

### Who will take informed consent? {26a}

Patients who are eligible to participate in the study receive information from the RR-TB clinic health staff and have the opportunity to discuss this information. All patients and the legal representatives of minors are asked to provide written informed consent to participate in the study, prior to undergoing any study-specific procedures. Patients are assured that their decision whether or not to participate in the study will not affect the quality of care they will receive. All patients who are ineligible for the study, refuse to be recruited, or withdraw after recruitment, are managed according to national guidelines without negative consequences for them.

### Additional consent provisions for collection and use of participant data and biological specimens {26b}

Patients who agree to participate in the study are asked to sign the consent form. They are informed that their sputum sample will be analysed in Niger and in Belgium and that the leftover of the sputum sample will be stored at the ITM for future research on tuberculosis for at least 10 years. Any future research needs to be approved by relevant Ethics Committees.

## Interventions

### Explanation for the choice of comparators {6b} and intervention description {11a}

The control arm relies on the Niger RR-TB treatment strategy. It relies on the national 9–11- month standard RR-TB STR, which includes moxifloxacin (MFX), CFZ, ethambutol (E), and pyrazinamide (Z) throughout, supplemented by a SLID (amikacin), prothionamide (PTO) and high-dose isoniazid (Hh; 10mg/kg) during the first 4 months (6 months if delayed conversion on smear microscopy at treatment month 4). The SLID is replaced with LZD in case of any hearing disturbance detected on audiometry (4-6 SLID-Hh-PTO-MFX-CFZ-E-Z/5 MFX-CFZ-E-Z, with LZD replacing the SLID in case of any hearing disturbance detected on audiometry, at baseline or during treatment).

In this control arm, the STR uses fluoroquinolone (moxifloxacin) as core drug, with both high bactericidal and sterilising activity. The SLID (or LZD if it replaces SLID) provides early bactericidal activity, and the other drugs add bactericidal or sterilising activity [16].

The experimental arm uses the WHO all-oral regimen, which contains LZD, high-dose isoniazid, prothionamide, high-dose levofloxacin (LFXh), BDQ, CFZ and pyrazinamide for 4 months (6 months if delayed conversion on smear microscopy at treatment month 4), followed by 5 months of treatment with high-dose levofloxacin, BDQ, CFZ and pyrazinamide (4-6 LZD-Hh-PTO-LFXh-BDQ-CFZ-Z/5 LFXh-BDQ-CFZ-Z).

BDQ and fluoroquinolone (levofloxacin) are used as core drugs, thus with high bactericidal and sterilising power. LZD, with good early bactericidal activity, protects BDQ and fluoroquinolone from the acquisition of resistance. The other drugs add bactericidal or sterilising activity [16].

### Criteria for discontinuing or modifying allocated interventions {11b}

A clinical committee may decide to modify the treatment regimen to start an individualised regimen in patients with specific problems. The most common situations for which the regimen may be discontinued include :

- Results of baseline DST may not be available before the start of treatment. If resistance to components of the treatment regimen is reported after the start of treatment it may be necessary to modify the treatment regimen taking into account DST results.
- Grade 3 or higher toxicity may lead to the temporary or permanent suspension of one or more drugs, which then may need to be replaced.
- Treatment failure (see the “Definition” section). In such patients, a new RR-TB treatment regimen is warranted. For the control arm, 4-6 Lzd-Hh-Bdq-Dlm-Cfz-Z/5 Bdq-Dlm-Cfz-Z will be used as re-treatment RR-TB regimen. For the experimental arm, the composition will be based on DST. The evaluation of re-treatment outcomes is not part of this study.

In patients with renal insufficiency the regimen is adapted as follows:

- If renal failure is present before the start of treatment, the SLID is replaced with LZD (similar approach as when audiometry abnormalities are present) and pyrazinamide is administered thrice weekly instead of daily.
- If during treatment with SLID the creatinine clearance is less than 90 ml/min, the SLID is prescribed twice or thrice weekly at 12–15 mg/kg; and ethambutol and pyrazinamide are given thrice weekly. If creatinine clearance is less than 60 ml/min the SLID will be replaced with LZD, while ethambutol and pyrazinamide are administered 3 times a week instead of daily.

Safety data collected during treatment with either the control or experimental regimen will be included in the safety analysis. Effectiveness data will be used from all patients enrolled on RR-TB treatment. However, if the regimen was changed during treatment to better fit the initial resistance pattern, such a patient will be excluded from the effectiveness analysis, as the patient had a contra-indication for the study regimen (see the exclusion criteria).

### Strategies to improve adherence to interventions {11c} {18b}

Avoiding the development of extensive drug resistance (resistance to any fluoroquinolone plus BDQ or LZD, in addition to RR-TB) is a priority. Since AE, particularly gastrointestinal, are frequent due to the high number of tablets, directly observed treatment (DOT) is compulsory throughout the duration of treatment. DOT is planned and implemented in close consultation with the patient and family so that an appropriate person to support the patient is identified, trained and supervised. All AE are clearly explained to patients and adequately treated and documented in their patient treatment cards. Ambulatory treatment is strongly recommended from the start of treatment (or as soon as possible) because it is more cost-effective than hospitalisation and it is often more acceptable to patients [17, 18]. Patient preferences and barriers such as long distances between the patient’s home and the health centre, transportation costs, incompatibilities of the centre’s opening hours with the service hours, etc., are assessed to adapt the service to the patient’s needs and improve adherence to treatment. Social support (reimbursement of daily transport costs for ambulatory treatment and nutritional support) is part of patient care. At least one visit is organised per patient, and as under routine care, the patient's home is visited by clinic staff at the beginning of the treatment, to better understand the patient’s living conditions, to be able to trace the home in a later phase, and to investigate those who are in close contact with the patient. Patients who delay for their appointment are contacted the same day or the next day, if possible first by phone. If this is not possible, a home visit is organised to explore why the patient was delayed, to prevent further irregularities, and to reinforce adherence to treatment.

### Relevant concomitant care permitted or prohibited during the trial {11d}

In RR-TB patients co-infected with HIV and on antiretroviral drugs we take into account the following measures:

- Tenofovir and SLID: strict monitoring of kidney function.
- Atazanavir and LZD: strict monitoring of the hemogram.
- Efavirenz (inducer of CYP3A4, thus decreases the concentration of BDQ) should not be used with BDQ.
- Protease inhibitors (inhibitors of CYP3A4, thus increase the concentration of BDQ) and BDQ can be used together with caution.
- Nevirapine and BDQ can be used together without problems

### Provisions for post-trial care {30}

In case of treatment failure or relapse sputum samples are sent to the ITM in Antwerp for DST. The National RR-TB Committee will construct a RR-TB retreatment regimen taking into account DST results. RR-TB retreatment will be provided while assuring the same level of quality of care as during the trial. This includes the continuation of the best possible TB treatment as long as needed, following national TB guidelines, and also care of any AE or complications.

### Outcomes {12}

Primary endpoint, as a measure of safety: any severe (grade 3–5) AE, with onset of symptoms before the end of treatment, and with the maximum grade measured up to 6 months after the end of treatment.

Secondary endpoints:

- As a measure of effectiveness: treatment outcome, with relapse-free success (cure or treatment completion without proof of relapse during 12 months of active post-treatment follow-up) as favourable outcome and treatment failure, relapse, lost to follow-up, and death as an unfavourable outcome.
- As a measure of adherence to the regimen: proportion of daily doses taken.

The definitions used to grade AE are based on French National Agency for Research on AIDS and hepatitis (ANRS) [19].

### Participant timeline {13}

Depending of the month of RR-TB diagnosis, as shown on the printed Xpert MTB/RIF result, the patient is enrolled on either the control or experimental treatment arm (a coin toss determines which regimen is used for which month). Each patient is treated for 9 to 11 months and is followed actively for 12 months after the end of treatment.

The inclusion assessment includes clinical, bacteriological, laboratory and additional tests described in Table 1.

**Table 1:** Enrolment and assessments

		Enrolment	Treatment phase (M = month)											Follow-up		Close-out
			M 1	M 2	M 3	M 4	M 5	M 6	M 7	M 8	M 9	M 10	M 11	MF 6	MF 12	
Eligibility screening		X														
Written informed consent		X														
Allocation		X														
Assessment	Clinical examination	X	X	X	X	X	X	X	X	X	X	X*	X*	X	X	
	Treatment adherence		X	X	X	X	X	X	X	X	X	X*	X*			
	Sputum smear	X (2)	X	X	X	X (2)	X (2)*	X (2)*	X	X	X (2)	X(2)*	X (2)*	X	X	
	Bacteriological culture	X		X		X		X			X	X*	X*	X	X	
	SL-LPA (FQ/Injectables)	X												X±	X±	
	Haemoglobin/CBC#, creatinine@, potassium@, liver enzymes	X	X	X	X	X	X*	X*								
	Visual acuity, test for peripheral neuropathy, Audiometry@	X	X	X	X	X*	X*									
	Chest X-ray	X									X	X*	X*			
	ECG§	X		X		X		X		X		X*	X*			
Safety outcome evaluation													X	X		
Treatment outcome evaluation																X

*If the intensive phase is prolonged

**±** DST carried out at baseline, after reversion (positive culture after conversion) during treatment, and if bacteriological culture is positive during the 12-month post-treatment follow-up (with storage of the baseline, treatment failure or relapse strain for genotypic analysis)

# To be done if the patient is taking linezolid

@ To be done if the patient is taking a second-line injectable drug

**§** A basic electrocardiogram (ECG) must be performed and additional ECGs done at weeks 1 and 2 after the start of treatment and then once a month during treatment. The ECGs must be repeated if necessary in the event of clinical suspicion of heart rhythm problems and conduction disorders, or other clinical signs (e.g. dehydration and electrolyte disorders)

Abbreviations: *CBC* complete blood count, *ECG* electrocardiogram, *FQ* fluoroquinolone, *M* month, *MF* month of follow-up after treatment completion, *SL-LPA* second-line line probe assay

Baseline sputum samples are systematically sent to the NRL and the ITM in Antwerp. However, as in any life-saving situation, treatment of drug-resistant tuberculosis is not withheld from a patient when DST results are not available. Hence the regimen may be modified once DST results become available {see 11b}.

All patients enrolled in the study undergo regular assessment of clinical and para-clinical parameters as described in Table 1. Monitoring procedures are adapted to fit the drugs used in either the control or experimental treatment arm (e.g. close monitoring of haemoglobin for patients taking LZD; monthly audiometry when a SLID is used).

Before starting treatment with linezolid, providers invite family members to collect blood bags to be able to do a timely blood transfusion in the event of bone marrow aplasia.

Every month sputum samples are collected for bacteriological monitoring using smear microscopy with auramine staining. In case of lack of conversion on smear microscopy at month four (two samples), the intensive phase is extended for one or 2 months maximally.

At month two, four, six and nine (10 or 11 if the intensive phase was prolonged with 1 or 2 months, respectively), samples are collected for culture. Post-treatment follow-up at 6 and 12 months after cure or treatment completion relies on smear microscopy and culture.

In the event of a treatment failure during treatment or relapse during the 12-month post-treatment follow-up, genotyping is done at the ITM in Antwerp on the baseline and failure/relapse sample to determine the strain identify and to distinguish between true treatment failure/relapse and reinfection.

At each visit, patients are asked about AE and all responses are recorded in the patient's file. Patients are advised to consult study staff in case AE occur between scheduled visits. If patients present with an AE or other problems requiring specific examinations between scheduled intervals, the frequency of monitoring and supervision will be adjusted.

- Patients on RR-TB treatment receive DOT during consultations at the TB clinic (thus physical site visits), which allows for regular screening of AE, with early referral to a medical doctor in case a severe AE is identified.
- Medical care is provided by nurses at the TB centres, with easy access to referral consultations by medical doctors and hospitalisation.
- Patients are informed to consult a TB nurse in case a new symptom emerges, or in case an existing symptom worsens.
- Patients living far from RR-TB Units stay in close contact by phone. Moreover, the nurse discusses whether visits can be spaced (for instance, from daily to weekly), and which days and hours convene best for the patient to come to the clinic.
- Transport costs are reimbursed, as in routine care.

### Sample size {14}

A total of 230 patients will be included prospectively to compare treatment arms (115 patients for each) in order to be able to identify a correlation between the treatment arms and the occurrence of any grade 3–5 AE, assuming that the “hazard ratio” for developing a grade 3-5 AE is 3.2 times higher among patients in the experimental treatment arm (WHO all-oral regimen) compared to the control arm (STR that contains either a SLID or LZD; 5% vs 15%; with power 0.8, alpha 0.05).

Justification of assumptions :

- Of the 153 patients already treated according to the Niger RR-TB treatment strategy, with LZD replacing the SLID in the STR in case of ototoxicity and with monthly audiometry, 6 (3.9%) had an AE of degree 3–5 [15]. For the calculation of the sample size, a “conservative” estimate of 5% was used.
- Of the 33 patients treated with linezolid instead of the SLID in case of ototoxicity, 6 (18.2%) had grade 3–5 AE [15]. A “conservative” estimate of 15% was used to calculate the sample size.

In addition to the prospective treatment arms, a retrospective cohort of 255 patients enrolled on treatment between 2017 and April 2021 will be used during an interim analysis (see interim analysis), if characteristics known to affect the treatment outcome of the retrospective cohort are not significantly different from the prospective arms, and in case the proportion with a grade 3-5 adverse event is not significantly different between the retrospective cohort and those prospectively treated using the Niger RR-TB treatment strategy (SLID-containing regimen, with LZD replacing the SLID in case of ototoxicity).

### Recruitment {15}

Prospectively participants diagnosed with RR-TB are enrolled by RR-TB clinic staff in inpatient or outpatient facilities.

## Assignment of interventions: allocation

### Sequence generation {16a}

**Fig. 1:**
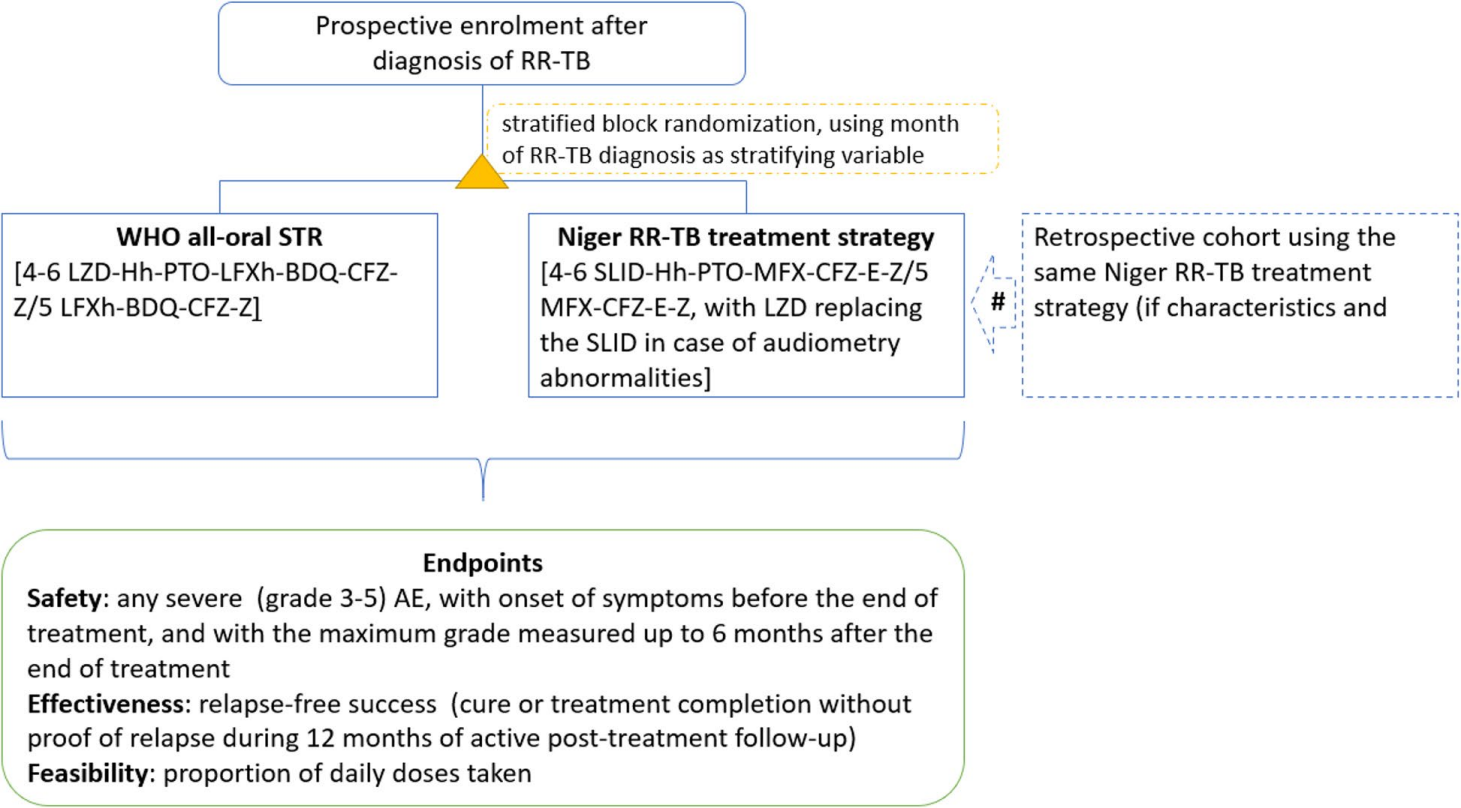
Flowchart showing study enrolment. # In addition to the prospective arms, a retrospective cohort of 255 patients enrolled on treatment between 2017 and April 2021 will be used during an interim analysis [if characteristics known to affect treatment outcome of the retrospective cohort are not significantly different from the prospective arms, and in case the proportion with a grade 3–5 adverse event is not significantly different between the retrospective cohort and those prospectively treated using the Niger RR-TB treatment strategy (SLID-containing regimen, with LZD replacing the SLID in case of ototoxicity)]

We randomise by monthly blocks. Depending of the month in which patients are diagnosed with RR-TB, patients with FQ-susceptible RR-TB are enrolled in either the experimental arm (WHO all-oral regimen, with BDQ and LZD) or control arm (STR that contains either a SLID or LZD) regimen. A coin toss determined which treatment arm is used for even (February, April, ...) or odd (January, March, ...) months for all RR-TB Units. The coin toss determined that odd months correspond to the experimental arm and even months to the control arm. The coin toss was done on 09 April 2021 by the principal investigator in the presence of the director of the NTP and all RR-TB units researchers to ensure that patients in the same clinic are treated with both the intervention during the trial study period. Following the coin toss, patients diagnosed in odd months are enrolled in the experimental arm, and those diagnosed in even months in the control arm (Fig. 1).

Data collected from patients who started RR-TB treatment between 1 January 2017 and 15 April 2021, when trial enrolment started, will be used for an interim analysis.

## Assignment of interventions: blinding

### Concealment mechanism and implementation; blinding {16b} {16c}{17a} {17b}

Laboratory staff who perform testing for the diagnosis of RR-TB are blinded to treatment assignment. They do not know which regimen is allocated to a given month. However, the RR-TB clinicians, patients (placebo is not feasible for TB regimens combining up to seven drugs, of which one is an injectable in one arm), and the researcher and statistician are not blinded for the regimens. When the patient arrives at the RR-TB clinic, depending of the month of RR-TB diagnosis the patient is either assigned to the experimental or control arm by the RR-TB clinic staff.

## Data collection and management

### Plans for assessment and collection of outcomes {18a}

Safety: Patients will be screened monthly by a person trained in the diagnosis and management of AE.

During the inclusion visit, pathologies associated with a high risk of adverse effects such as diabetes, kidney and liver failure, malnutrition, HIV infection, alcohol and drug abuse, etc. should be identified and recorded. The underlying causes of adverse effects will be identified and treated.

AE are classified according to their severity using ARNS scale (Table 2) and noted in the patient’s file.

**Table 2:** Severity rating of adverse effects

Degree	Description
Degree 1: Slight abnormality	Mild or transient discomfort, without limitation of usual daily activity; does not require medical intervention or corrective treatment.
Grade 2: Moderate abnormality	Partial limitation of usual daily activity; medical intervention or corrective treatment required. No need to interrupt treatment.
Grade 3: Severe abnormality	Limitation of usual daily activity; requires medical intervention and corrective treatment, possible hospitalisation. It may be necessary to stop the responsible medication temporarily until the symptoms disappear.
Grade 4: Life threatening or permanent injury	Very limited activity; requiring medical intervention and corrective treatment, almost always in a hospital environment. It may be necessary to stop the responsible drug permanently.
Grade 5: Death	

Grade 1 adverse events will only be recorded in the patient's file without further action; those of grade 2 often require a medical intervention with auxiliary drugs

Any grade 3–5 AE are classified as severe AE

All AE will be reported in the patient treatment card. When an AE occurs, the investigator responsible for the care of the patient will first assess and grade the event. If it is graded 4–5, or if other serious AE (SAE) criteria (such as hospitalisation) apply, then a “severe AE form” will be completed and sent to the principal investigator and the relevant pharmacovigilance authority (Fig.1).

The relationship between a severe AE and drugs used in the STR can only be carried out by the physician in charge of the study. He will analyse and consider possible other causes of the observed AE before concluding that they are due to a particular anti-TB drug. The categories showing the level of causality categories include “definite,” “probable,” “possible,” “unlikely,” “not related,” and “unclassifiable” and are detailed in Table 3.

**Table 3:** Scale for attribution of assessing drug relationship with AE occurrence

Category	Definition
Definite	Events occurring within a timely manner after administration of the drug(s); that are known sequela to the administration of the drug(s) and follow a previously documented pattern of reaction, but for which no other explanation is known. This category applies to AEs that the principal investigator believes are incontrovertibly related to the treatment.
Probable	Any event occurring in a timely manner after administration of the drug(s); that follows a known pattern of reaction to the drug(s); and for which no other explanation is known. This category applies to AEs that, after careful medical consideration at the time they are evaluated, are believed with a high degree of certainty to be related to the drug(s).
Possible	Any event occurring in a timely manner after administration of the drug(s) that does not follow a known pattern of reaction and for which no other explanation is known. This category applies to AEs that, after careful medical consideration at the time they are evaluated, are considered unlikely to be related but that cannot be ruled out with certainty.
Unlikely	In general, this category can be considered applicable to those AEs that, after careful medical consideration at the time they are evaluated, are considered to be unrelated to administration of the drug(s).
Not related	Any AEs for which there is evidence that an alternative aetiology exists or for which no timely relationship exists to the administration of the drug(s) and the AEs do not follow any previously documented pattern. This category applies to those AEs that, after careful medical consideration, are clearly and incontrovertibly due to causes other than the drug(s).
unclassifiable (insufficient data to assess)	There is insufficient information about the AE to allow for an assessment of causality.

Effectiveness: Outcomes will be assessed during treatment and, in case of cure or treatment completion, at the end of 12 months post-treatment follow-up

*Therapeutic success*: Composite result corresponding to the combination of “cured” + “treatment completed” (= treatment success) without relapse during the 12-month follow-up period

Note: this result can also be defined as “recovery without relapse”.

*Cured*: A patient with bacteriologically confirmed RR-TB who has completed 9-11 months of treatment without evidence of treatment failure AND at least 2 consecutive cultures at least 30 days apart are negative in the continuation phase and not followed by a positive culture result during treatment and post-treatment follow-up.

*Treatment completed*: A patient who has completed 9 to 11 months of treatment with the regimen with no evidence of treatment failure BUT not fulfilling the definition of “cured”.

*Treatment failure*: The treatment is interrupted or it is necessary to permanently change the treatment regimen of at least two anti-tuberculosis drugs due to:

- A lack of bacteriological conversion of sputum after 6 months of treatment, or
- A reversion of the sputum bacteriological culture after 7 months of treatment in a patient with a previously negative culture, or
- Evidence of additional drug resistance acquired in the treatment regimen of the study in patients with either treatment failure or relapse, and without proof of a between baseline and recurrence genotypically different strain, or
- AE leading to the modification of at least two anti-TB drugs in the treatment regimen

*Deceased*: A patient who has died during treatment from any cause.

*Lost to follow-up:* A patient whose treatment has been interrupted for 2 or more consecutive months.

*Not evaluated*: A patient for whom no treatment outcome is assigned (this includes cases “transferred out of the study area” to another treatment unit and whose treatment outcome is unknown or cannot be assessed)

*Relapse:* A patient who has been treated for RR-TB, has been declared “cured” or “treatment completed” and is diagnosed with another episode of confirmed RR-TB with rifampicin-resistant bacilli, within one-year follow-up, and without proof of a between baseline and recurrence genotypically different strain.

*Reinfection:* Recurrence (treatment failure or relapse) with proof of a between baseline and recurrence genotypically different strain.

*Withdrawn*: A patient is withdrawn from treatment for a reason other than treatment failure (e.g. resistance to second-line basic drugs, the patient’s informed consent is withdrawn or other reasons) and referred back to the standard treatment programme for routine care.

For the purpose of the effectiveness analysis, relapse-free success will be the favourable outcome. Unfavourable outcomes include death, failure, lost to follow-up during treatment and relapse (patients withdrawn will be excluded).

Adherence: Adherence will be assessed by the number of patients who are receiving the experimental treatment arm among all eligible patients, compared to the number of patients on the control treatment arm. Patients are considered adherent when 90% of the treatment doses were taken. Adherence is assessed based on the information on the treatment cards, measured over the total treatment period.

*Processing time*: The cumulative delay between sample collection and the start of RR-TB treatment.

*Registration rate:* The number of patients who started treatment among those with confirmed RR-TB.

### Data management {19}

For this pragmatic trial, we rely on routinely used data sources, such as the laboratory register, RR-TB treatment register, and the patient’s RR-TB treatment card. Data are imputed in Excel sheets, used for routine reporting since 2008 [8]. All source documents, as well as the consent forms, are archived at the RR-TB clinics. Routinely conducted quarterly supervision visits ensure data completeness. These visits are performed by the national RR-TB focal point, part of the National RR-TB Committee.

Data from these sources will be encoded in a national database in Excel (already is in place), and updated regularly by the research assistants, supervised by the principal investigator.

### Confidentiality {27}

Care and treatment are provided by NTP clinicians supported by DF staff. Patients will not be contacted by staff other than those who are also responsible for routine services. NTP clinicians at the RR-TB unit are responsible for ensuring appropriate storage of all study-related documentation.

All patient data will be pseudonymised in the database by means of a unique study number assigned to the participants. Treatment cards, lab results, and registers will only be accessible to NTP and DF staff involved in RR-TB care and their supervisors, all trained in confidentiality. The supervisors are also involved in the present RR-TB research.

### Plans for collection, laboratory evaluation and storage of biological specimens for genetic or molecular analysis in this trial/future use {33}

Baseline sputum samples are collected from all patients enrolled. Additional sputum samples are collected from patients with treatment failure or relapse. Sputum samples (outcome sample) will be stored in alcohol and sent to the supranational ITM laboratory in Antwerp to determine the resistance profile using Deeplex®-MycTB Deeplex for deep target sequencing (Deeplex; Genoscreen, Lille, France). Deeplex results obtained from the outcome sample will be compared with those obtained from the baseline sample to identify acquired resistance and to distinguish true failure or relapse (same strain) from reinfection (different strain). In case no Deeplex results are obtained, spoligotyping will be used to show the identity of the strain.

## Statistical methods

### Statistical methods for primary and secondary outcomes {20a}

#### Primary analysis

The proportion of patients with grade 3–5 AE will be compared between the experimental treatment arm and the control arm using a chi-square test (or exact Fisher test). A Cox regression model will be developed to measure the association between enrolment in either the experimental or control arm and the incidence of a grade 3–5 AE. All patients who started treatment will be included. The start of follow-up time will be the date of start of treatment. The end of follow-up time will be the date an AE was graded as grade 3–5. In patients without AE, the date of the end of treatment will be used if they were cured or completed treatment, or the date of the last visit in case the patient was lost to follow-up before ending the treatment, or the date of death in case the patient died during treatment.

#### Secondary analysis

By treatment arm, safety will be calculated as the proportion of all patients started on treatment and without any severe AE (safety endpoint). By treatment arm, treatment effectiveness will be calculated as the proportion of patients with a favourable outcome among all patients with either a favourable or unfavourable outcome. By treatment arm, adherence will be calculated as the proportion of patients who took at least 90% of the doses of RR-TB treatment. Cohorts will be compared using the chi-squared test. Bivariable and multivariable logistic regression models will be used to estimate the association between having an unfavourable treatment outcome and treatment arm (experimental vs control; the variable of interest), adjusted for potential confounding factors such as age, sex, HIV status, type of tuberculosis, bacillary load, extent of disease, body mass index (BMI) and initial resistance pattern. Factors associated (*p*-value <0.1) with having an unfavourable outcome will be included in the multivariable model, which then will be simplified by backwards elimination until all remaining factors have a *p*-value <0.05 (treatment arm, the variable of interest, will not be removed).

An additional Cox regression model will be developed to estimate the association between the variable “exposure to SLID vs LZD” and having a grade 3–5 AE probably or definitively related to either the SLID (ototoxicity) or LZD (haematological disorders, optic neuritis, neurological disorders). The variable “exposure to the injectable vs linezolid” will be treated as a time-dependent covariate, as described previously [15].

Analyses will be carried out with Stata software (version 16.1, College Station, TX).

### Interim analyses {21b}

As soon as the safety data for 80 prospectively included patients are available, an intermediate analysis will be carried out to evaluate whether a significant association exists between the treatment arm (experimental vs control) and the occurrence of the safety endpoint (any grade 3–5 AE), using Cox regression, as described above for the primary analysis.

As the Niger approach to RR-TB treatment (SLID-containing STR with LZD replacing the SLID in case of audiometry abnormalities) was already successfully used and as data were collected rigorously, using the same procedures that will be applied in the trial, interim analyses will also include the retrospective cohort if the characteristics of the cohort are not significantly different from the prospective cohort with both treatment arms (the chi-square test will be used to compare HIV status, bacillary load, extent of TB disease and body mass index), and in case the proportion with a grade 3–5 adverse event is not significantly different between the retrospective cohort and those prospectively treated using the Niger RR-TB treatment strategy. To assess whether data from the retrospective cohort can be used, besides age and gender, characteristics known to predict safety (BMI, co-morbidities) and effectiveness (baseline bacillary load, baseline resistance profile) will be compared between the retrospective and prospective cohorts.

If the interim analysis shows a significant difference between the experimental arm and the control arm in terms of the primary endpoint before the total sample size is reached, study enrolment will be discontinued.

In case enrolment continued after the first interim analysis, and as soon as safety data are available for 160 patients enrolled in the trial, a second interim analysis will be conducted, using the same approach as used during the first interim analysis.

### Methods for additional analyses, e.g. to handle missing data (e.g. subgroup analyses) {20b}{20c}

Patients lost to follow-up during treatment will have an unfavourable treatment outcome. In case data are missing for an independent categorical variable, the missing indicator method will be used, and a category “missing” will be added.

### Plans to give access to the full protocol, participant level-data and statistical code {31c}

Once completed, de-identified trial data will be shared upon request and approval by the National Tuberculosis Program of Niger and Damien Foundation Niger.

## Oversight and monitoring

### Composition of the coordinating centre, trial steering committee, data monitoring committee {5d}{21a}

The trial is sponsored by DF. The trial is implemented by the NTP through the RR-TB National Committee and DF. ITM provides DST and research expertise. The treatment monitoring group consists of the principal investigator (from DF), the technical RR-TB referent from DF, the focal point of the Niger RR-TB National Committee, and a co-investigator from ITM. The treatment monitoring group will meet to discuss the results of the interim analysis and decide whether enrolment should continue, or not (see the interim analysis).

### Adverse event reporting and harms {22}

All AEs that occur during the study are documented and followed until resolution or stabilisation. All AE will be recorded in the patient’s file and in a database by type, degree and month of occurrence. All SAE (grade 4–5 AE or AE requiring hospitalisation) deemed related to one or more investigational product(s) and considered unexpected with the use of such products are reported to National Regulatory Authorities. All other severe AEs are reported in an annual safety report. In addition, safety data is reviewed quarterly by the RR-TB National Committee, the members of which have expertise in clinical trials, RR-TB, pharmacology. Line listings of all reported serious AE’s will be sent on a yearly basis to the National Pharmacovigilance Unit and ethics review bodies.

### Frequency and plans for auditing trial conduct {23}

Regular data review and data monitoring and cleaning for quality control is performed quarterly in accordance with GCP guidance requirements by the principal investigator and national RR-TB focal point.

### Plans for communicating important protocol amendments to relevant parties (e.g. trial participants, ethical committees) {25}

In case the procedures would have to change, an amendment will be developed and submitted to the relevant ethics review bodies.

### Dissemination plans {31a}

The results will be communicated through abstract presentations at scientific conferences and publications in peer-reviewed journals. The results of the intermediate analysis can be used for abstracts or manuscripts, prior to the publication of the results of the primary analysis.

## Discussion

In Niger, during the past 10 years, the RR-TB treatment strategy was highly successful, with 83% relapse-free cure [8]. RR-TB patients are treated with a SLID-containing STR. The STR’s SLID is replaced by LZD in patients with ototoxicity. Audiometry is done at baseline and on a monthly basis during treatment. As any audiometry abnormality serves as indication for switching to LZD, not a single RR-TB patient developed severe hearing loss since 2017 [15]. BDQ is safeguarded for the rare patients with treatment failure or relapse after a first STR, leaving not a single patient without a treatment option [8]. Since 2020 WHO recommends constituting all-oral STR, thus replacing the SLID with bedaquiline. Considering the highly successful Niger RR-TB treatment strategy, it could only be modified if a rigorous evaluation, relying on the unbiased comparison between the all-oral WHO STR and the Niger RR-TB treatment approach would show benefits in terms of safety and effectiveness.

To maximally avoid bias, patients had to be randomly assigned to either an experimental arm, using the all-oral WHO STR, or the control arm, relying on the Niger RR-TB treatment approach. To increase the feasibility of conducting a RCT, embedded in routine activities of all four RR-TB clinics in Niger, we adapted our trial design. First, instead of randomising patients on an individual basis to either the newly recommended all-oral WHO STR or the control arm, relying on the SLID-containing STR for most and with LZD replacing the SLID in case of any ototoxicity, we randomised by monthly blocks, whereby the regimen used changes every month. This approach simplifies the work of the clinicians running the RR-TB clinics and also overseeing this trial. With monthly blocks, major causes of bias are prevented as patients are not assigned by the clinician to one or another treatment arm. Allocation to a month is defined by the date of RR-TB diagnosis, which seems a better option than the date of RR-TB treatment start, as treatment start could be delayed by the clinician depending on how he perceives both regimens.

We preferred to not use a before-and-after design, or stepped-wedge design, with the all-oral regimen replacing the presently used SLID-containing regimen because of limits of this design. A randomised clinical trial randomising individual patients seemed not feasible. RR-TB trials are challenging. Heterogeneous patterns of drug resistance and adverse drug reactions lead to a variability in regimens, which interferes with the head-to-head comparison between regimens. The relatively low number of patients diagnosed with RR-TB results in a longer enrolment period, compared to studies on drug-susceptible TB. Moreover, the treatment duration is long [20]. Due to these challenges, it can take 5 years or more between RR-TB trial design and having results available [21]. Therefore, WHO treatment regimen recommendations are largely based on data from observational studies, usually showing data for a single treatment strategy, and expert opinion [12]. To overcome these challenges, TB treatment trials should embrace bold and creative approaches that can produce high-quality evidence on treatment safety and efficacy [21]. We therefore used an approach that would be feasible for the NTP to provide high-level evidence on the one hand, but also to inform local and international guidelines in time.

Second, we will conduct interim analyses to stop enrolment early in case the primary safety endpoint would be significantly more frequent in one of both arms. For the interim analysis, we will also consider including data from a retrospective cohort of patients treated in precisely the same way as in the control arm, in terms of treatment and monitoring procedures, including consent procedures and prospective collection of safety and treatment response data. We will verify whether the characteristics of the retrospective cohort are significantly different from those of the RCT cohort and whether the proportion with a grade 3–5 adverse event is significantly different between the retrospective cohort and those prospectively treated using the Niger RR-TB treatment strategy (SLID-containing regimen, with LZD replacing the SLID in case of ototoxicity). If not different, data from the retrospective cohort will be used. As such we aim to avoid a too long enrolment period and important delays between this study and informing national and international treatment guidelines. A previous analysis showed a higher rate of grade 3-5 adverse events in a small observational cohort of patients treated with a LZD-containing STR compared to the SLID-containing STR. With our trial, we therefore hypothesise that the risk of severe adverse events is higher among patients treated with the newly recommended WHO all-oral regimen, with LZD for all, compared to the control arm, with a STR that contains either SLID or LZD. If indeed, the new WHO all-oral regimen shows to cause more adverse drug reactions, it would ethically not be justified to expose more patients than needed to this regimen, especially considering the exceptionally good results obtained with the control arm in our setting.

The findings of our trial will immediately inform the implementation of RR-TB treatment by the Niger NTP. All RR-TB sites are involved, thus findings are generalisable to the reality of Niger. The NTP director is involved as a co-investigator and thus can guide the implementation of recommendations informed by our findings. Our findings will also inform the international debate on the balance between striving towards a RR-TB treatment regimen that is both safe and effective.

Our trial has important strengths. Findings will be generalisable to the Niger context, where RR-TB care is standardised, using the same procedures in all RR-TB treatment units, which all are participating in this trial. The design used is feasible for routine RR-TB care, using data collection and monitoring procedures in place for over a decade. As such we expect prospectively collected data to be reliable and complete. We anticipate that potential confounding bias will be overcome by random allocation with monthly blocks. An alternative design that would also have been feasible, e.g. a before-after cohort study, certainly would have resulted in a higher risk of confounding bias. To not extend the duration of the trial beyond what is justified, and considering that the RR-TB programme is very stable, we will also add already collected data on the RR-TB treatment strategy in the interim analysis, unless characteristics of the retrospective cohort differ from the prospective cohort.

The study design also has some weaknesses. Monthly block randomisation is still more vulnerable to confounding bias than individual randomisation. Therefore, using a multivariable regression, we will aim at correcting for any effect of known predictors of safety and effectiveness outcomes. Nevertheless, some residual confounding may still not be adjusted for. Secondly, the incidence of adverse events caused by e.g. second-line injectable drugs and/or linezolid varies by region. Therefore our safety findings may not be applicable to all settings. We hope that our creative study design will inform (RR-)TB trials in other countries, as updated WHO recommendations with a “very low certainty in the estimate of the effect” are translated to national policy and practice.

## Trial status

Recruitment for the study began at all four sites on 15 April 2021. Recruitment is expected to be completed on 14 April 2024 (3 years). The last patient enrolled will complete treatment on 14 January 2025 and post-treatment follow-up for one year on 15 January 2026.

## Data Availability

Anonymized study data may be made available on request following study closure.

## Abbreviations

aDSM: Active drug safety monitoring
AE: Adverse event
BDQ: Bedaquiline
CFZ: Clofazimine
DF: Damien Foundation
DLM: Delamanid
DOT: Directly observed treatment
DST: Drug susceptibility test
RIF: Rifampicin
RR-TB: rifampicin-resistant tuberculosis
SAE: Severe adverse event
SLID: Second-line injectables drugs
SL-LPA: Second-line line probe assay
STR: Short treatment regimen
TB: Tuberculosis
WHO: World Health Organisation
Z: Pyrazinamide
E: Ethambutol
FQ: fluoroquinolone
Hh: High-dose isoniazid
ITM: Institute of Tropical Medicine
LFXh: High-dose levofloxacin
LPA: Line probe assay
MFX: Moxifloxacin
NRL: National Referral Laboratory
NTP: National tuberculosis program
PTO: Prothionamide
RCT: Randomised clinical trial
